# A Rare Case of MRSA Pericarditis with Expanding, Purulent Pericardial Effusion Leading to Uremic Kidney Failure from a Right, Necrotic Toe

**DOI:** 10.1155/2022/7041740

**Published:** 2022-10-29

**Authors:** Justin Brilliant, Diep Edwards, Ritu Yadav, Jana Lovell, Lena Mathews

**Affiliations:** Johns Hopkins Hospital, Baltimore, MD, USA

## Abstract

Purulent pericarditis is an extremely rare entity with only a few reported cases so far. This condition deserves prompt diagnosis because of its significant mortality rate if left untreated. A 76-year-old man with a past medical history of coronary artery disease (CAD) with percutaneous coronary intervention (PCI) to the left anterior descending artery (LAD) and right circumflex artery (RCA), ischemic cardiomyopathy with moderately reduced ejection fraction (EF 45-50%), peripheral artery disease (PAD), COVID-19 pneumonia complicated by fibrotic lung disease (on 3 liters of home oxygen), type-2 diabetes mellitus (T2DM), hypertension (HTN), hyperlipidemia (HLD), and chronic kidney disease (CKD) stage III presented with complaints of pleuritic chest pain and shortness of breath. On hospital day 1, he was afebrile and hemodynamically stable with physical exam remarkable for bibasilar crackles and dry gangrene of his right first toe. He developed progressive altered mental status, hypotension, oliguric renal failure, and respiratory distress on hospital day 6. On exam at this time, he had an elevated jugular venous distension (JVD) of 12-14 cm water, pericardial friction rub with decreased heart sounds, and orthopnea; all were consistent with cardiac tamponade clinically. An electrocardiogram (EKG) showed new ST elevations in leads I, II, and aVL with ST depression in aVR and V1 with only mild elevation in troponin I to 0.07 ng/mL. A transthoracic echocardiogram (TTE) was done on hospital day 7 and showed a moderate sized pericardial effusion with inferior vena cava (IVC) enlargement but no atrial collapse, ventricular collapse, IVC collapse, or respiratory variation in the mitral and tricuspid inflow velocities. Blood cultures grew methicillin-resistant *Staphylococcus aureus* (MRSA) on hospital day 6, and he was started on intravenous (IV) vancomycin. The differential diagnosis for his enlarging pericardial effusion included purulent pericarditis, uremic pericarditis, or hemorrhagic effusion. He had urgent diagnostic and therapeutic pericardiocentesis with removal of 350 milliliters of fluid. The pericardial fluid was cloudy, tan-brown with a gram stain showing gram-positive cocci in clusters and cultures growing MRSA, which confirmed the diagnosis of purulent pericarditis secondary to MRSA infection. After the pericardiocentesis, his blood pressure, respiratory distress, and renal failure improved. The source of the bacteremia was from osteomyelitis of his gangrenous, right toe with bone biopsy growing both MRSA and *Streptococcus anginosus*. He underwent toe amputation for definitive source control. He was discharged on hospital day 24 with a plan to complete 6 weeks of IV vancomycin.

## 1. Introduction

Pericarditis is the most common disease of the pericardium worldwide, which can be a manifestation of an underlying systemic disease or an unrelated primary process. In developed countries, most pericarditis is idiopathic with a presumed viral etiology [1]. Purulent pericarditis, a localized bacterial infection of the pericardial space characterized by gross pus, only accounts for less than 1% of all cases with MRSA being the most commonly isolated organism [1]. This organism colonizes the nasopharynx, perineum, and skin, and it can migrate into the circulatory system and lead to hematogenous spread if the cutaneous barrier is disrupted or damaged. The diagnosis of purulent pericarditis from MRSA is extremely rare, with only a few case reports thus far [2–8]. However, the mortality rate for untreated patients can approach 100% but decreases to 40% in those patients who are appropriately treated with antibiotics and source control [9]. Furthermore, the coincidental diagnosis of uremia may lead to further pericardial injury to facilitate the accumulation of fluid. In this report, we present a case of MRSA pericarditis with purulent pericardial effusion and development of uremic renal injury complicated by a right necrotic toe.

## 2. Case Presentation

We present a 76-year-old man with a past medical history of CAD who had undergone multiple PCIs with drug-eluting stents to the LAD (18 and 12 years prior to admission) and RCA (2 years prior to admission) complicated by ischemic cardiomyopathy with moderately reduced ejection fraction (EF 45%), PAD, CKD stage III, T2DM, HTN, and HLD. Fifteen months prior to admission, he had developed viral pneumonia from SARS-CoV-2 complicated by chronic hypoxemic respiratory failure with fibrotic lung disease requiring supplemental oxygen of 3 liters since that time. Our patient presented with acute onset of substernal, pleuritic chest pain exacerbated by inspiration and accompanied by shortness of breath on the morning of presentation. The chest pain was noted to be very different from his prior myocardial infarctions and was not relieved by sublingual nitroglycerin. The patient described shortness of breath as if “someone threw a bucket of water” at him. His home pulse oximeter showed that at that time of pain, his oxygen saturation was in the 90 s, and his heart rate varied from 50 s to 130 s. The patient also reported right great toe gangrene that developed after a traumatic toenail clipping with worsening discoloration over the last month, but overall, he was able to bear weight and did not experience fevers, chills, or toe pain.

**Figure 1:**
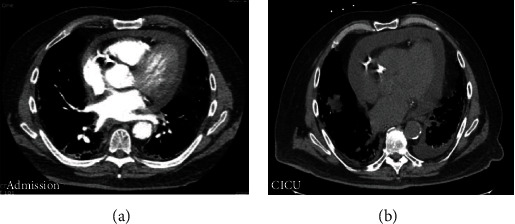
CT chest on hospital day 1 (a) and hospital day 7 (b). The pericardial effusion had expanded from a largest diameter of 1.5 cm (a) to 2.0 cm (b) along with increase in left pleural effusion.

Vital signs on admission showed a temperature of 98.2°F, heart rate of 91/min, blood pressure of 147/81 mm of Hg, and respiratory rate of 18/min with oxygen saturation 98% on 3 L supplemental oxygen (home requirement). Physical exam was notable for bibasilar crackles, JVD 9 cm water, and dry gangrene of his right first toe. His chest pain subsided, and he remained chest pain free in the emergency department; serial troponins were negative. His initial labs were notable for white blood cell count (WBC) 11.31 K/*μ*L, hemoglobin (Hb) 9.1 g/dL, platelets 289 K/*μ*L, creatinine 1.6 mg/dL (baseline 1.3-1.4 mg/dL), blood urea nitrogen (BUN) 23 mg/dL, international normalized ratio (INR) 1.0, D-dimer 1.19 g/L, T4 1.25 nmol/L, *troponin* *I* < 0.04 *ng*/*mL*, negative urine toxicology, erythrocyte sedimentation rate (ESR) 87 mm/hr, C-reactive protein (CRP) 25.9 mg/L, lactate 1.2 mmol/L, negative testing for SARS-CoV-2 via nasopharyngeal swab (Labcorp PCR test), negative testing on a respiratory viral pathogen nasopharyngeal swab (Labcorp PCR test), and negative blood cultures. EKG showed normal sinus rhythm with no ST changes greater than 1 mm or T wave inversions. Computed tomography angiography (CTA) showed chronic fibrotic lung disease, new small pericardial effusion of 1.5 cm in its largest diameter, but no pulmonary embolism (Figure 1). Initial TTE showed an EF of 45-50%, with small, posterior pericardial effusion. There was mild concentric hypertrophy of the left ventricle (LV) with moderate hypokinesis of the mid and apical inferolateral, mid to distal anteroseptal, apical, apical lateral, and mid anterolateral segments along with akinesis of the basal inferoseptal segment; no significant valvular dysfunction or vegetation was seen. He was admitted for acute decompensated heart failure and underwent IV diuresis with furosemide. He developed new onset atrial fibrillation with rapid ventricular response on day 2 for which he was started on an IV heparin drip and an IV amiodarone drip. He converted to sinus rhythm on hospital day 5.

**Figure 2:**
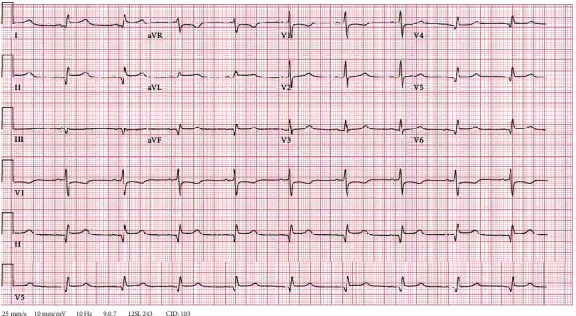
EKG on hospital day 7 with enlarging pericardial effusion. Normal sinus rhythm with new 2 mm ST elevations in leads I and II, 1 mm elevation in aVL, submillimeter elevations in V5-V6, and 1 mm ST depression in aVR and V1.

**Figure 3:**
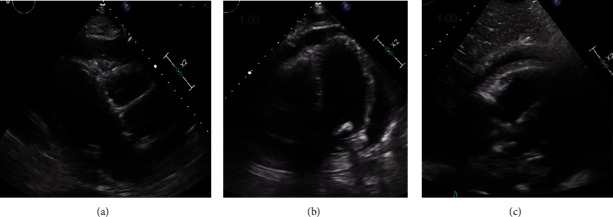
Parasternal long (a), apical 4-chamber view (b), and subxiphoid view (c) via echocardiography of expanding pericardial effusion on hospital day 7. No right atrial or right ventricular collapse or respiration variation in mitral or tricuspid inflow was seen. The inferior vena cava (IVC) was enlarged without collapse.

On day 6, our patient developed oliguric acute kidney injury with creatinine peaking at 4.4 mg/dL with BUN 102 mg/dL. He also became hypotensive with oliguria no longer responsive to diuresis at the time, which had required the initiation of continuous venovenous hemodialysis (CVVHD). However, our patient did not tolerate ultrafiltration because of low blood pressure. The administration of broad-spectrum antibiotics including IV vancomycin and IV cefepime was initiated due to concern for septic shock. However, with elevated JVD of 12-14 cm H20, hypotension, and evidence of ongoing renal failure, our team was concerned about obstructive shock in the setting of cardiac tamponade. He had persistent shortness of breath and developed worsening pleuritic chest pain, orthopnea, and a friction rub on exam. A repeat CT scan showed that the pericardial effusion enlarged from 1.5 cm to 2 cm in its greatest width (Figure 1). An EKG at the time showed normal sinus rhythm with new 2 mm ST elevations in leads I and II, 1 mm elevation in aVL, submillimeter elevations in V5-V6, and 1 mm ST depression in aVR (Figure 2). Troponin I was only mildly elevated to 0.07 ng/mL. On hospital day 7, an expanding pericardial effusion was seen on a limited TTE measuring 2.5 cm posteriorly in subcostal view with enlarged IVC but no evidence of tamponade without findings of atrial collapse, ventricular collapse, IVC collapse, or respiratory variation in mitral or tricuspid inflow (Figure 3). With concern for hemorrhagic effusion, anticoagulation for atrial fibrillation was held. Given the characteristics of his chest pain, pericardial friction rub on exam, EKG findings, and evidence of pericardial effusion on TTE, our patient met 4/4 criteria for acute pericarditis based on the most recent 2015 European Society of Cardiology (ESC) guidelines for diagnosis of pericardial disease [10].

**Figure 4:**
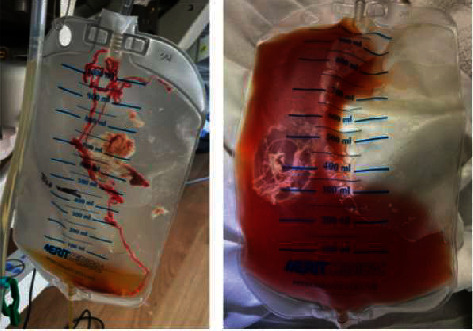
Pericardiocentesis with immediate removal of 350 cc cloudy, tan-brown, sanguinous fluid.

Our patient's blood cultures from hospital day 6 returned positive for MRSA, for which the patient's antibiotics was narrowed to only vancomycin after two days of receiving cefepime. On day 8, he was transferred to the cardiac critical care unit for therapeutic pericardiocentesis; 350 mL of tan-brown cloudy thin fluid was drained under pressure, and a pericardial drain was left in place (Figure 4). He had immediate improvement in his hypotension and renal failure, and thus, did not require any additional dialysis. Postprocedure TTE confirmed complete drainage. Fluid pH was 6.9 with WBC 18,500/mm^3^ with 95% neutrophilic predominance, red blood cells of 13000/mm^3^, and gram stain showed gram positive cocci, raising concern for purulent pericarditis. Pericardial fluid cultures grew MRSA. The pericardial drain was left in place until hospital day 12 after no additional drainage for 48 hours and only a trace pericardial effusion seen on repeat TTE without echocardiographic evidence of tamponade. A magnetic resonance imaging (MRI) scan of the right foot performed on hospital day 11 showed an increased T2 signal of the distal phalanx of the great toe with corresponding T1 hypointense signal in the bone marrow compatible with acute osteomyelitis. His right necrotic great toe up to the metatarsophalangeal joint was amputated by vascular surgery on hospital day 18 for primary source control. The cultures from the resected toe and the surrounding margin of resection grew MRSA (and *Streptococcus anginosus*) that were both susceptible to vancomycin confirming the diagnosis of osteomyelitis. Given renal recovery to a creatinine of 1.3 (baseline for patient) with robust urine output following pericardial drainage, the decision was made to continue treatment with intravenous vancomycin (rather than daptomycin or linezolid) for 6-weeks after his repeat blood cultures became negative on day hospital day 8.

Regarding the patient's peripheral artery disease complicated by right great toe gangrene, noninvasive imaging demonstrated impaired perfusion to the right lower extremity. He was taken to the cardiac catheterization lab for a right lower extremity angiogram and diagnostic aortogram with long segment balloon angioplasty of the peroneal and anterior tibial arteries before amputation of necrotic right toe. As the patient was progressing towards medical readiness for discharge, he developed the acute onset of chest discomfort. A 12-lead ECG demonstrated new inferior ST segment elevations with reciprocal lateral changes as compared to a prior tracing. The patient was transported to the cardiac catheterization laboratory for emergent coronary angiography and possible primary PCI. The patient was found to have chronically occluded right coronary artery (RCA) and diffusely diseased distal left anterior descending (LAD) artery. PCI was not attempted.

## 3. Discussion

Both purulent pericarditis secondary to MRSA bacteremia and uremia are distinct entities that can lead to a rapidly evolving pericardial effusion [1, 11]. These two conditions can be difficult to differentiate because of similarly presenting symptoms such as acute chest pain, particularly in the recumbent position, pericardial friction rub on examination, and possibly wide-spread ST segment elevation [1, 12, 13]. Our patient met all of the criteria (4/4) for diagnosis of acute pericarditis given his history with physical examination, EKG, and echocardiography. However, early diagnosis and treatment of purulent pericarditis from MRSA with sufficient source control are critical because of the downstream complications of pericardial tamponade, septic shock, pericardial abscess formation, and constrictive pericarditis [10]. With greater than one-third of patients with purulent pericarditis secondary to MRSA initially presenting with a lack of bacteremia and a high risk of pericardial injury in the setting of uremia, identification of the etiology can be difficult, but pericardial drainage and source control is critical to avoid the aforementioned complications due to delay in intervention [14].

Purulent pericarditis from any bacterial organism is very rare and accounts for less than 1% of all cases of pericarditis with the majority being idiopathic from a presumed viral etiology [1]. The risk factors for development of purulent pericarditis include prior thoracic surgery, CKD, immunosuppression, alcohol use, and undiagnosed neoplasm [10]. The most commonly cited organisms include *Staphylococci*, *Streptococci*, and *Enterococci* with anaerobic species (e.g., *Prevotella* and *Peptostreptococcus*) seen more with concomitant infection in the mediastinum or neck [15]. Fungal pathogens should be considered for patients at risk for candidemia including parenteral hyperalimentation, prolonged antibiotic therapy, or steroid administration [16]. For immunosuppressed patients on chemotherapy or diagnosed with acquired immune deficiency syndrome (AIDS) from human immunodeficiency virus (HIV), *Mycobacterium tuberculosis* involvement of the pericardium has been documented [10, 15]. Purulent pericarditis most commonly develops from either hematogenous spread from a distant source or direct spread from a pleural empyema or pneumonia [10]. In addition, perforating injury, surgery, myocardial abscess formation, and endocarditis are less common etiologies [15]. However, for cases of purulent pericarditis from MRSA, hematogenous spread has been the most commonly cited means of seeding the pericardium with only a few cases reporting osteomyelitis as a documented source [17, 18]. An expedited TTE with pericardiocentesis should be done for any clinical suspicion for an enlarging pericardial effusion because of the high prevalence of cardiac tamponade (84%) noted in a recent systematic review [14]. Cultures from the pericardial fluid should be sent for bacterial, fungal, and tuberculosis studies to confirm the diagnosis [10]. Purulent pericarditis from MRSA has a 100% mortality rate if untreated because of resulting multiorgan failure from sepsis, but with adequate drainage and antibiotic treatment, mortality rate decreases to 30% [19]. Therefore, prompt initiation of antibiotics that target MRSA along with placement of either a pericardial drain (such as our patient), pericardial window, pericardiotomy, or even surgical pericardiectomy is recommended since the recurrence of effusion is common and had been noted to be 30% in reported cases [1, 10]. Even with attempted source control with the use of antibiotics and drainage of pus in the pericardial space, the feared complications of septic shock, reaccumulation of pus with potential abscess formation, and constrictive pericarditis can occur and have been reported in 15.4%, 30.8%, and 3.5% of reported cases, respectively [14, 19].

In addition to purulent pericarditis, uremia alone can lead to the accumulation of toxic metabolites and nitrogenous waste products that are highly toxic to the pericardium leading to inflammation, fibrin deposition, and adhesion [10]. In severe cases, a pericardial effusion can also develop; however, large volume fluid accumulation in the pericardial space may be partially related to platelet dysfunction in patients with renal failure. The diagnosis of uremic pericarditis requires a strong clinical suspicion. Aside from pleuritic chest pain, objective data include diffuse ST and T-wave elevation, elevated cardiac biomarkers, and confirmatory echocardiogram; however, those EKG findings may not always occur because epicardial injury can be uncommon [20]. Initial treatment includes prompt initiation of dialysis as most patients (87%) resolve rapidly with intensive dialysis [21]. Urgent pericardiocentesis is only recommended within 7-14 days or emergently if there is evidence of cardiac tamponade [22]. The precipitants of cardiac tamponade in uremic pericarditis include hypovolemia, paroxysmal tachyarrhythmia, and concomitant epicardial injury [23].

The prompt initiation of antibiotic treatment for suspicion of purulent pericarditis, in conjunction with drainage and source control with pericardiocentesis, is critical and should be directed against the most likely pathogens based on patient-specific factors. An empiric regimen includes a combination of an antistaphylococcal antibiotic (e.g., vancomycin) along with either an aminoglycoside (e.g., gentamicin) or cephalosporin (e.g., ceftriaxone and cefepime) for gram-negative coverage; the addition of a fluoroquinolone (e.g., ciprofloxacin and levofloxacin) can be considered if no rapid improvement in clinical signs of infection is seen to cover for atypical infections such as *Legionella* and *Mycoplasma* [15, 24]. Empiric therapy should be continued until signs and symptoms of infection are improving and then narrowed after the diagnosis is confirmed from the pericardial fluid with the specific pathogen isolated. There are no specific guidelines on the duration of treatment for purulent pericarditis, but treatment should last for at least 2-4 weeks contingent upon the adequacy of drainage with definitive source control and the antimicrobial susceptibilities of the isolated strain of microbe(s) [15]. The duration of antibiotics should also be based on additional sources of infection for which guideline-based courses of therapies exist such as concomitant osteomyelitis and endocarditis where antibiotic duration would be extended to 6 weeks. Finally, no current recommendation exists for serial monitoring of the pericardial effusion via echocardiography after completion of treatment unless new symptoms or lab abnormalities arise to suggest recurrence of infection.

In regard to the complications of purulent pericarditis, notably cardiac tamponade, and constrictive pericarditis, invasive procedures such as pericardiocentesis, subxiphoid pericardiotomy with fibrinolytic therapy, and pericardiectomy are considered depending on the degree of cardiac compression, chronicity, and recurrence [10]. Although urgent pericardiocentesis with effective drainage is a class I recommendation for diagnosis and treatment of purulent pericarditis, transcatheter intrapericardial fibrinolysis has been shown to decrease rate of constriction by 38.3% [25]. Furthermore, subxiphoid pericardiotomy with fibrinolytic therapy and even surgical pericardiectomy may be considered (class IIa recommendation) for more definitive management to allow for complete drainage of the effusion, manual lysis of adhesions, and decreased risk of developing constrictive pericarditis [10]. A subxiphoid pericardiotomy is preferred over a surgical pericardiectomy when surgery is too high of a risk (i.e., large, recurrent pericardial effusions or cardiac tamponade) or for a more palliative approach [10]. However, the communicating passage from the pericardial space may close off and repeated, and loculated effusions may occur. A pericardiectomy, therefore, allows for complete removal and can be the definitive therapy for either recurrent episodes of purulent pericarditis or constrictive pericarditis with the removal of each constricting pericardial layer [26]. Additional studies are needed to clarify the timing, delivery, and efficacy of transcatheter and intrapericardial fibrinolytic therapies in relation to surgical pericardiectomy in patients with purulent pericarditis.

Ultimately, our patient was discharged from the hospital without any complications on IV vancomycin to complete a 6-week duration of treatment for osteomyelitis of his right foot. Purulent pericarditis from MRSA was suspected early in our patient's admission because of an expanding pericardial effusion with clinical evidence of cardiac tamponade. The subsequent drainage of pus with pericardiocentesis and drain placement along with a source of the MRSA isolated from the right toe explained the hematogenous spread to the pericardium. Although our patient's uremic renal injury could have contributed to his worsening pericardial effusion, uremic pericarditis was not a consideration for his initial presentation given the presence of a small effusion before his clinical decompensation, lack of improvement following dialysis, and prompt resolution of symptoms following pericardial drainage and antibiotic treatment. With early initiation of antibiotics against MRSA and successful amputation of his right necrotic toe that grew MRSA confirming osteomyelitis, our patient was discharged on IV vancomycin to complete a total 6-week duration of therapy because of residual infected bone present after resection. However, our patient was transitioned from vancomycin to linezolid to complete the final 5 days of his treatment course given an increase in his creatinine from 1.3 to 2.6. Following his completed course of antibiotics, his right foot has healed without any complications, creatinine has improved close to baseline at 1.7, and a repeat TTE showed stable cardiac function with trace pericardial effusion similar in size prior to discharge.

## 4. Conclusion

MRSA purulent pericarditis is an extremely rare diagnosis. In this report, we present a case of MRSA pericarditis in a patient with type 2 diabetes mellitus and peripheral artery disease complicated by a right necrotic toe who developed acute, oliguric renal failure with an enlarging pericardial effusion. The patient underwent diagnosis and primary source control with pericardial drainage and toe amputation in addition to a total, six-week course of antibiotics with activity against MRSA. Given the significant morbidity and mortality of MRSA pericarditis, early recognition and treatment of bacterial pericarditis are critical. Further studies are warranted to guide diagnostics and optimize therapies in high-risk populations such as those with multiple cardiovascular and renal comorbidities.
